# Preconception care: it’s never too early

**DOI:** 10.1186/1742-4755-11-73

**Published:** 2014-10-02

**Authors:** Sunni L Mumford, Kara A Michels, Natasha Salaria, Pilar Valanzasca, José M Belizán

**Affiliations:** Epidemiology Branch, Division of Intramural Population Health Research, Eunice Kennedy Shriver National Institute of Child Health and Human Development, National Institutes of Health, 6100 Executive Blvd. 7B03, Rockville, MD 20852 USA; BioMed Central, London, UK; Institute for Clinical Effectiveness and Health Policy, Buenos Aires, Argentina

## Abstract

The preconception window has been recognized as one of the earliest sensitive windows of human development, and interventions that focus on this period have the potential to affect not only pregnancy but long term outcomes as well. The journal *Reproductive Health* has published a supplement entitled ‘Preconception Interventions’ which includes a series of systematic reviews regarding the impact of public health interventions during the preconception period on maternal and child health. These articles describe the role that poor preconception health plays in creating health disparities across the globe. The reviews highlight our current understanding (or lack thereof) regarding how both maternal and paternal preconception health and knowledge shapes the long-term health of not only children, but of families, communities, and nations. Researchers and healthcare workers should take particular note of these interventions, as the preconception time period may be as important as the pregnancy and post-pregnancy periods, and is critical in terms of bridging the gap in the continuum of care, particularly for adolescents.

## Editorial

Healthy lifestyles during pregnancy are known to be associated with improved pregnancy outcomes for both mothers and offspring. As such, much attention has been placed on designing effective prenatal care guidance, and considerable research has been done to identify appropriate interventions to improve maternal and child health during the prenatal period. However, the importance of exposures during the preconception period is becoming increasingly apparent; this time may not be too early to start intervening to improve the health of couples and their families.

The preconception window has been recognized as one of the earliest sensitive windows of human development [1], and taken together with the developmental origins of health and disease (DOHaD) paradigm [2], highlights the importance of the preconception period for optimizing health. Not only does the preconception period have the potential to affect immediate maternal and child health, but exposures during this sensitive time can have effects on downstream reproductive and developmental endpoints, as well as urological and gynecological health and later onset disease.

Thus there is a need for improved evidence-based preconception guidance and interventions. This need has been recognized by several organizations, and in particular, the Centers for Disease Control recently launched a campaign to raise awareness regarding the importance of preconception care, regardless of a woman’s pregnancy intentions [3].These types of preconception interventions are essential as a large proportion of births are unintended [4] and that even among couples attempting pregnancy, there is some evidence that women don’t change their behaviors [5].

However, there are notable difficulties to research during the preconception period. In particular, there is a need to recruit both those who are planning and not planning their pregnancies. In addition, not all couples that attempt to conceive become pregnant, and couples that do become pregnant may be reluctant to participate in research studies during such a stressful time. It is also important to recognize that under a life course approach, evidence is needed regarding preconception exposures for both females and their male partners across their lifetimes. Despite these challenges, preconception recruitment is feasible [6–11] and such data is needed to inform interventions and preconception guidance. These interventions and guidance have the potential to carry through preconception, pregnancy, and across the lifespan—thus underscoring the need for evidence-based approaches.

In response to this need, the journal *Reproductive Health* is pleased to publish a series of systematic reviews about the impact of public health interventions during the preconceptional period on maternal and child health [12–17]. These articles describe the role that poor preconceptional health plays in creating health disparities across the globe—most notably by showing how few studies are performed among populations in regions like Africa and the Middle East. The reviews highlight our current understanding (or lack thereof) regarding how both maternal and paternal preconception health and knowledge shapes the long-term health of not only children, but of families, communities, and nations.

There are several important components to preconception care, including lifestyle factors (e.g., smoking, alcohol, caffeine, weight), vaccinations, chronic disease management, accessibility of contraception, and environmental exposures. Each of these areas is addressed in detail in the supplement, offering a comprehensive assessment of the available evidence. The first article in the supplement reviews the impact of preconception counseling on maternal and child health outcomes [12]. Here, Dean and colleagues also provide a new definition for preconceptional care that accounts for the realistic length of time needed to impact health behaviors and chronic conditions: preconception care is “any intervention provided to women and couples of childbearing age, regardless of pregnancy status or desire, *before* pregnancy, to improve health outcomes for women, newborns and children [12]”. The authors stress the importance of maintaining inter-conception health within this definition. The next review describes the impact of increasing birth spacing, preventing teen pregnancy, and improving access to post-abortion care; the role of delayed childbearing is also explored through the literature on advanced maternal age [13]. The last four reviews focus on preconceptional interventions that improve nutrition and decrease infections, chronic disease, and medicinal and chemical exposures [14–17]. These authors are able to show that much of what we know about improving pregnancy health or health in general applies to the preconceptional period. For example, folic acid and multivitamin use initiated during this period and pre-pregnancy weight loss (among overweight individuals) can improve neonatal health [14]. These studies further emphasize the need for public health messages that address healthy lifestyle behaviors across the lifespan.

As you read through these articles, you will also note marked gaps in this literature. As mentioned previously, there are many regions not represented in preconception research. The effectiveness of many public health interventions is uncertain in areas that often have poor preconceptional and child health. In many of these reviews, the authors remark that study heterogeneity and the disparate methods for identifying exposures and reference groups also makes it difficult to understand what interventions are successful. The authors cite the measurement of caffeine intake across studies as one source of confusion and show that the paucity of research on pharmacodynamics and pharmacokinetics both before and during pregnancy makes pre-pregnancy recommendations on the use of these substances questionable [16, 17]. The need to close the gap in terms of appropriate interventions to benefit in underserved areas should be a priority for future research.

With information regarding the importance of preconception, the next step is to successfully implement these interventions. The last two articles of this supplement discuss the qualities of successful public health interventions aimed at improving preconceptional health and how they might be implemented on a local and national scale [18, 19]. The ultimate goal of these interventions should be to improve health outcomes, rather than place “blame” on mothers by focusing on their behaviors as a source of problems [19]. Finding ways to provide education and health services to women that empower them to “be proactive in matters concerning their own health” and which do not limit their autonomy is a necessity [18, 19]. Yet, education and care targeted toward men and boys is just as important for improving child and preconceptional health [12, 19]. Multi-component education programs, especially those for adolescents, are described as providing great benefits (e.g., those that provide leadership skills, mentoring, and improve health literacy) [19]. As Lassi et al. adeptly say, “Knowledge gives a teenager the tools for rational reasoning and judgment. Knowledge helps a teen make better choices. Knowledge gives you options” [19].

This series of articles makes clear that preconceptional health should not only be considered as a special research topic within maternal and child health—but that the welfare of children, reproductive aged individuals, and those transitioning between these periods is a priority and critical for prevention of morbidity and mortality in later life and in subsequent generations. This supplement is our call to arms. Clinicians, epidemiologists, health services researchers, economists, educators, behavioral scientists, pharmacologists, lawyers, and social justice activists in the public, private, and academic sectors—there is a message here for each of you.

We understand the impact that poor maternal and child health has on the long-term health of individuals, populations, and economies. Many of the solutions for improving these health outcomes are supported by a strong evidence base (e.g., multivitamin use, weight loss, smoking cessation, condom use), though there is still a need to improve the evidence available to better inform preconception health interventions. Many of these solutions may require us to act and intervene earlier than we may have realized. It’s time to ask the hard questions: How do we develop local, realistic, and manageable interventions that can benefit both women and men throughout their lives, regardless of their pregnancy intentions? And how do we do this in places where women’s autonomy is threatened, food and medicines are scarce, healthy behaviors are culturally scrutinized, or paternalism from healthcare providers is vilified? As preconception interventions have the potential to impact pregnancy and health across the lifespan, it could be argued that it is never too early to intervene.
